# Real-World Effectiveness of Risankizumab in Crohn’s Disease: Outcome Data for an IL-23 Inhibitor from the Middle East

**DOI:** 10.1093/crocol/otaf062

**Published:** 2025-11-09

**Authors:** Mohamed Nasir Alzaabi, Thaer Khaleel Swaid, Hosameldin Abdelrahman Ahmed, Maryam A Alahmad, Nadeen Mamon Omar, Kishore Kumar Chitra Kumar, Enas Fouad Ahmed, Talha A Malik, Mohammed Nabil Quraishi

**Affiliations:** Department of Gastroenterology, Sheikh Shakhbout Medical City, Pure Health, Abu Dhabi, United Arab Emirates; Department of Gastroenterology, Sheikh Shakhbout Medical City, Pure Health, Abu Dhabi, United Arab Emirates; Department of Gastroenterology, Sheikh Shakhbout Medical City, Pure Health, Abu Dhabi, United Arab Emirates; Department of Gastroenterology, Sheikh Shakhbout Medical City, Pure Health, Abu Dhabi, United Arab Emirates; Department of Gastroenterology, Sheikh Shakhbout Medical City, Pure Health, Abu Dhabi, United Arab Emirates; Department of Gastroenterology, Sheikh Shakhbout Medical City, Pure Health, Abu Dhabi, United Arab Emirates; Department of Gastroenterology, Sheikh Shakhbout Medical City, Pure Health, Abu Dhabi, United Arab Emirates; Department of Gastroenterology, Mayo Clinic Arizona, Arizona, 85250, United States of America; Department of Gastroenterology, Sheikh Shakhbout Medical City, Pure Health, Abu Dhabi, United Arab Emirates; Institute of Cancer and Genomic Sciences, University of Birmingham, Birmingham, B15 2TT, United Kingdom; College of Medicine and Health Sciences, Khalifa University of Science and Technology, Abu Dhabi, United Arab Emirates

## Abstract

**Introduction:**

Crohn’s disease (CD) in the Middle East is often aggressive, yet regional patients are underrepresented in pivotal trials for new therapies. This study is the first real-world evaluation of risankizumab’s effectiveness, safety, and dose optimization in a predominantly Emirati cohort with moderate-to-severe CD.

**Methods:**

This prospective cohort included 60 UAE patients with moderate-to-severe CD initiating risankizumab. Endpoints included clinical remission (CDAI <150) and biochemical remission (normalised C-reactive protein <5 mg/L and faecal calprotectin <250 µg/g), assessed post-induction (weeks 12-20) and at maintenance (10-14 months). Logistic regression models were used to identify predictors of remission and the need for dose intensification.

**Results:**

The median age was 33 years, and 61.7% were advanced therapy (AT)-exposed. Post-induction, clinical remission was achieved in 46.7% of patients, with significantly higher rates in AT-naïve vs AT-exposed patients (69.6% vs 32.4%, *P* = .011). Biochemical remission was achieved in 53.3% overall, again favouring the AT-naïve group (78.3% vs 37.8%, *P* = 0.005). At maintenance (*n* = 48), clinical and biochemical remission rates were 72.9% and 70.8%, respectively. Later-line risankizumab use was a significiant predictor of lower odds of post-induction remission (OR 0.27, *P* = .001). In AT-exposed patients, prior ustekinumab exposure was associated with lower remission rates (OR 0.11, *P* = .021) and a higher likelihood of dose intensification (OR 5.67, *P* = .045). Dose intensification recaptured clinical response in 62.5% (5/8) of patients. No new safety signals were identified.

**Conclusion:**

This first Middle Eastern real-world study confirms risankizumab is effective and safe for complex CD and supports its use across different treatment lines.

## Introduction

Crohn’s disease (CD) is a chronic, immune-mediated inflammatory condition of the gastrointestinal tract that often requires long-term treatment with advanced therapies to achieve and sustain remission.^1^ While therapeutic options have expanded, real-world data on newer agents like IL-23 inhibitors are crucial, particularly from regions with unique disease characteristics. IL-23 has emerged as a key therapeutic target due to its central role in promoting intestinal inflammation via the Th17 pathway.^2^^,^^3^ Risankizumab, a selective monoclonal antibody targeting the p19 subunit of IL-23, has demonstrated superior efficacy over placebo in phase 3 induction and maintenance trials,^4^^,^^5^ with significant long-term outcomes.^6^

Despite this promising evidence base, pivotal trials for IBD therapies—including those evaluating IL-23 inhibitors—have largely been conducted in Western populations, with minimal representation from the Middle East and North Africa (MENA) region.^7^ This underrepresentation is a significant knowledge gap, as emerging data suggest that CD in the MENA region, including the United Arab Emirates (UAE), can present with a particularly aggressive phenotype. Studies from the UAE have highlighted a high burden of CD, often diagnosed at a younger age, with notable rates of complicated disease behaviour such as stricturing (18%-27%), penetrating (18%-30%), and particularly high rates of perianal disease (34%-47%) at diagnosis or early in the disease course.^8^^,^^9^ This contrasts with some Western cohorts where such complications often develop over a longer period. Such aggressive presentations underscore the urgent need for effective therapies and real-world outcome data for this unique, under-explored patient population. Potential pharmacogenomic differences and distinct environmental exposures in the region may also influence therapeutic response and long-term outcomes, further limiting the generalizability of existing trial data.

Given the distinct, often aggressive, CD phenotype observed in the Middle East and the paucity of regional data on IL-23 inhibitors, there is an urgent need for high-quality, real-world evidence. This study presents the first prospective evaluation of risankizumab in Crohn’s disease patients from the Middle East (UAE), significantly expanding on our previously published pilot data by including a larger cohort, longer follow-up, and analysis of predictors and dose optimization.^10^ The primary objective was to evaluate the real-world effectiveness of risankizumab for inducing and maintaining clinical and biochemical remission in this cohort, which is predominantly of Emirati background and reflective of the complex local patient population. Secondary objectives included describing baseline characteristics differentiating advanced therapy (AT)-naïve and AT-exposed patients, assessing the impact of prior therapy exposure on outcomes, identifying predictors of remission and the need for dose intensification, and reporting safety data. By providing robust data on effectiveness, safety, and treatment optimization in this unique regional context, particularly for patients with aggressive disease, this study aims to contribute essential evidence to guide the positioning of risankizumab within Middle Eastern IBD treatment paradigms.

## Methods

This was a real-world observational cohort study with a hybrid prospective and retrospective design, conducted at a tertiary IBD centre in the United Arab Emirates. The study is anchored by the SSMC IBD registry, which was established in mid-2022 to prospectively collect detailed datasets including patient-reported outcomes, disease biomarkers, and treatment persistence in IBD patients under active follow-up at the centre. Adult patients with a confirmed diagnosis of moderate-to-­severe Crohn’s disease who initiated on risankizumab between January 2022 and June 2025 were included. For all patients, data following their enrolment into the registry were collected prospectively at predefined intervals. For the small subset of patients who initiated therapy before the registry was formally launched, baseline and early induction data were abstracted retrospectively from electronic health records. Inclusion criteria required completion of at least one full induction course (intravenous 600 mg monthly for three months). Patients received maintenance dosing of subcutaneous 360 mg every 8 weeks following successful completion of induction. Patients were excluded from the final analysis if therapy was discontinued prior to post-induction review for non-medical reasons (eg, loss of insurance, non-compliance), or if key baseline or outcome data were missing. Specifically, relevant data included baseline disease activity scores (CDAI) and inflammatory markers (CRP, faecal calprotectin). Reporting of this study adheres to the STROBE (Strengthening the Reporting of Observational studies in Epidemiology) guidelines.^11^

Baseline demographics, disease phenotype, laboratory parameters, and prior therapy exposure were recorded at risankizumab initiation. Patients were stratified as advanced therapy (AT)-naïve or AT-exposed based on previous biologic or small molecule use. Disease location, behaviour, and age at diagnosis were classified per the Montreal system. Laboratory values—including haemoglobin, CRP, albumin, and faecal calprotectin—were recorded within 4 weeks pre-treatment. Clinical activity was assessed using CDAI by the gastroenterologist, and endoscopic severity was evaluated using the SES-CD by the performing endoscopist when available. Outcomes were assessed at two timepoints: post-induction (weeks 12-20) and maintenance (10-14 months); selected to align with established long-term follow-up points in clinical trials. The primary clinical outcome was post-induction clinical remission, defined as CDAI <150. Clinical response at both timepoints was defined as a ≥ 100-point reduction in CDAI from baseline. Biochemical remission at both timepoints was defined as normalisation of both CRP (<5 mg/L) and faecal calprotectin (<250 µg/g). Endoscopic response during maintenance was defined as a ≥ 50% reduction in SES-CD from baseline.

All analyses were conducted in R (version 4.3.1).^12^ Comparative analyses were performed between advanced therapy (AT)-naïve and AT-exposed groups. Continuous variables were summarised as medians with interquartile ranges (IQR), and categorical variables as frequencies with percentages. Between-group comparisons used Wilcoxon rank-sum for continuous data and Chi-squared or Fisher’s exact tests for categorical data. To identify predictors of outcomes, multivariable logistic regression models were used. For the primary outcome of post-induction clinical remission, the model incorporated disease duration prior to risankizumab, baseline haemoglobin, baseline albumin, baseline CDAI, baseline SES-CD, disease behaviour, presence of perianal disease, and risankizumab line of therapy. In the AT-exposed subgroup, the impact of specific prior therapies (anti-TNF, ustekinumab, vedolizumab, and upadacitinib) was assessed using separate univariate models. A separate logistic regression model evaluated prior ustekinumab exposure as a predictor for dose intensification. Variables for inclusion in the models were selected a priori based on their known or potential clinical relevance in predicting treatment outcomes in Crohn’s disease.

## Ethical Considerations

The study was approved by the Institutional Review Board at SSMC—REC number SSMCREC-425. All data used in this analysis were anonymized to protect patient confidentiality, and informed consent was waived due to the retrospective nature of the study. The study was conducted in compliance with the Declaration of Helsinki.

## Results

**Figure 1.**
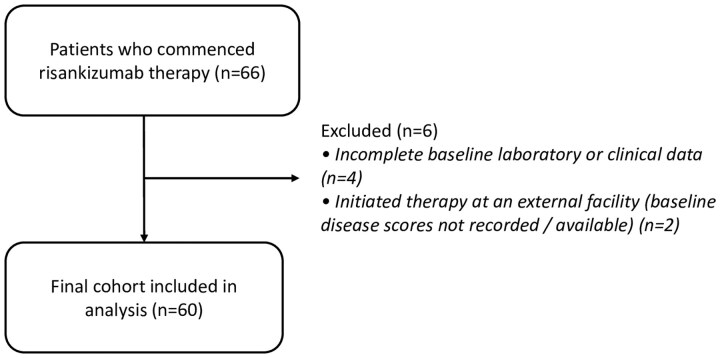
Flowchart of patient disposition. The diagram illustrates the inclusion and exclusion of patients, detailing the process from the initial number of patients who commenced risankizumab to the final cohort included in the analysis.

From an initial cohort of 66 patients who commenced risankizumab, six were excluded from the final analysis: four due to incomplete baseline laboratory or clinical data, and two who initiated therapy at an external facility where a baseline CDAI was not recorded. This resulted in a final cohort of 60 patients with moderate-to-severe Crohn’s disease who completed induction therapy and were included in the analysis. The patient selection process is detailed in Figure 1. This cohort was predominantly of Emirati background, with 54 of 60 patients (90%) being UAE nationals, reflecting the local population served by this tertiary referral centre.

Detailed baseline characteristics are shown in Table 1. Twenty-three patients (38.3%) were advanced therapy (AT) naïve, while 37 (61.7%) had prior exposure to one or more biologic or small molecule agents. The median age at risankizumab initiation was 33 years (IQR 26-43), and the cohort was 40% female (24/60). Regarding disease phenotype, most patients had ileocolonic involvement (67%), followed by isolated ileal disease (28%), and a minority with purely colonic disease (5.0%). Inflammatory disease behaviour was most common overall (57%), but more prevalent in the AT-naïve group (78%) than in AT-exposed patients (43%; *P* = .015). Stricturing (24%) and penetrating (32%) disease behaviour were more common among AT-exposed patients compared to AT-naïve (13% and 8.7%, respectively). Perianal disease was also significantly more frequent in the AT-exposed group (43% vs 13%; *P* = .044). AT-exposed patients had a significantly longer median prior disease duration at treatment initiation (86 months [IQR 47-163] vs 7 months [IQR 2-54]; *P* < 0.001) and higher baseline median CDAI (280 [IQR 250-320] vs 198 [IQR 117-260]; *P* = .006) compared to AT-naïve patients. The median duration of risankizumab therapy was 13 months (IQR 9-18) across the cohort. Corticosteroids were used at baseline for only 4 patients due to rapid access to biological therapies and infusion unit slots.

**Table 1.** Summary of baseline characteristics.

	All patients, *N* = 60^a^	Advanced therapy naive, *N* = 23^a^	Advanced therapy exposed, *N* = 37^a^
Age (years)	33 (26, 43)	36 (25, 45)	32 (26, 37)
Female gender	24 (40%)	13 (57%)	11 (30%)
Prior disease duration (months)	62 (17, 141)	7 (2, 54)	86 (47, 163)
Haemoglobin (g/dL)	129 (115, 140)	122 (114, 130)	130 (120, 142)
CRP (mg/L)	8 (2, 18)	7 (1, 16)	8 (2, 21)
Albumin (g/L)	35.0 (31.8, 40.0)	37.0 (32.5, 40.5)	34.0 (32.0, 39.0)
Faecal calprotectin (µg/g)	350 (95, 1,198)	334 (42, 2,220)	366 (103, 1,145)
CDAI	250 (160, 300)	198 (117, 260)	280 (250, 320)
SES-CD	8.0 (4.8, 11.3)	8.0 (4.0, 13.0)	8.0 (5.0, 10.0)
Disease location			
Colonic	3 (5.0%)	1 (4.3%)	2 (5.4%)
Ileal	17 (28%)	9 (39%)	8 (22%)
Ileo-Colonic	40 (67%)	13 (57%)	27 (73%)
Upper GI involvement	1 (1.7%)	0 (0%)	1 (2.7%)
Disease behaviour			
Inflammatory	34 (57%)	18 (78%)	16 (43%)
Penetrating	14 (23%)	2 (8.7%)	12 (32%)
Stricturing	12 (20%)	3 (13%)	9 (24%)
Perianal disease	19 (32%)	3 (13%)	16 (43%)
Exposed to only 1 prior AT			12
Exposed to 2 or more prior AT			25
Prior exposure to Anti-TNF			31 (83.8%)
Prior exposure to Ustekinumab			24 (64.9%)
Prior exposure to Vedolizumab			10 (27%)
Prior exposure to Upadacitinib			3 (8.1%)

a Median (IQR); *n*/*N* (%).

**Figure 2.**
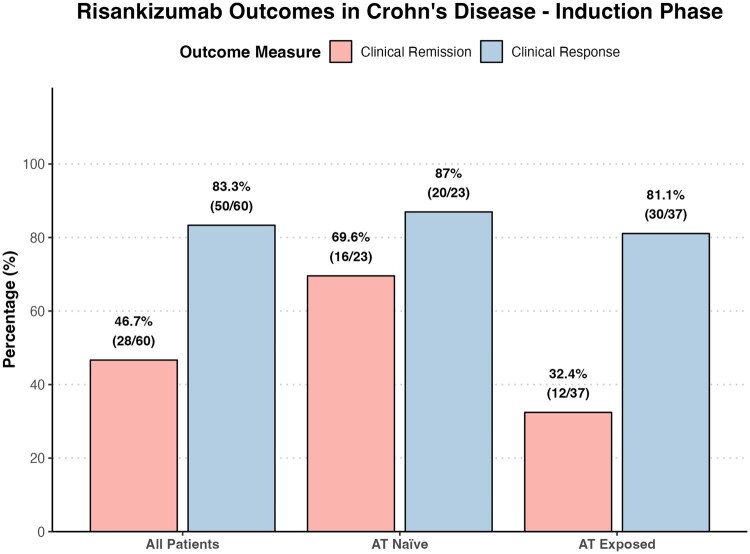
Risankizumab outcomes (clinical remission and clinical response) at induction (week 12-20) in all patients, advanced therapy (AT) naïve patients, and AT exposed patients.

Post-induction and maintenance outcomes are summarised in Table 2. At post-induction follow-up (week 12 to 20), 28 of 60 patients (46.7%) achieved clinical remission (CDAI <150), while 52 patients (86.7%) met criteria for clinical response (≥100-point CDAI reduction) (Figure 2). Clinical remission was significantly more frequent among AT-naïve patients (69.6%) compared to those previously exposed to advanced therapies (32.4%; *P* = .011). Although clinical response was numerically higher in the AT-naïve group (87% vs 81.1%), the difference was not statistically significant (*P* = .73). Biochemical remission, defined as normalization of both CRP and faecal calprotectin, was achieved in 53.3% of patients overall (32/60) and was also significantly higher in AT-naïve patients (78.3%) compared to AT-exposed patients (37.8%; *P* = .005).

**Table 2.** Outcomes post induction (week 12 to 20) and at maintenance (month 10 to 14).

	All patients	Advanced therapy naive	Advanced therapy exposed	*P*-value
Post induction clinical remission	28/60 (46.7%)	16/23 (69.6%)	12/37 (32.4%)	.0112
Post induction clinical response	50/60 (83.3%)	20/23 (87%)	30/37 (81.1%)	.727
Post induction biochemical remission	32/60 (53.3%)	18/23 (78.3%)	14/37 (37.8%)	.00535
Maintenance clinical remission	35/48 (72.9%)	16/18 (88.9%)	19/30 (63.3%)	.0922
Maintenance biochemical remission	34/48 (70.8%)	16/18 (88.9%)	18/30 (60%)	.0473
Maintenance endoscopic response	28/44 (63.6%)	15/18 (83.3%)	13/26 (50%)	.0493

**Figure 3.**
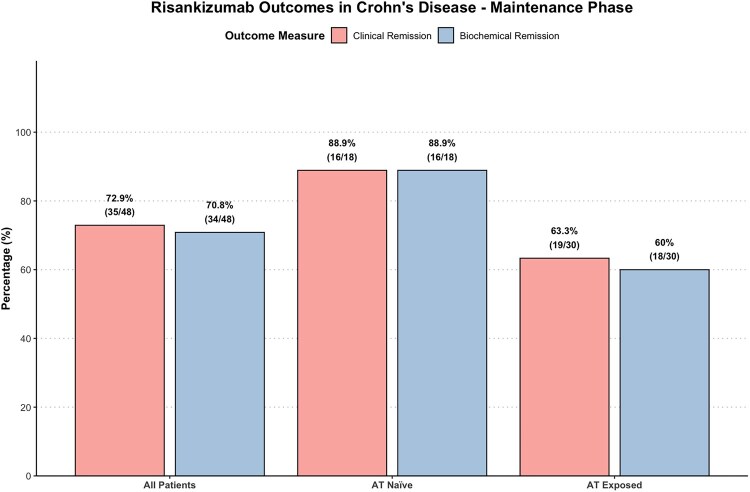
Risankizumab outcomes (clinical remission and biochemical remission) at maintenance (month 10-14) in all patients, advanced therapy (AT) naïve patients, and AT exposed patient.

Of the 60 patients who completed induction, two discontinued therapy due to primary non-response. Of the remaining cohort, maintenance outcomes were assessed in the 48 patients for whom at least 12 months of follow-up data were available (other remaining patients had not yet reached this timepoint). Among 48 patients with at least 12 months of follow-up, 72.9% achieved clinical remission (35/48) and 70.8% (34/48) achieved biochemical remission. During maintenance, clinical remission was numerically more frequent in the AT-naïve group (88.9%) compared to the AT-exposed group (63.3%), though this difference did not reach statistical significance (*P* = .092; Figure 3). However, maintenance biochemical remission was significantly more frequent in the AT-naïve group (88.9%) compared to the AT-exposed group (60%; *P* = .047). ­Endoscopic response, defined as ≥50% reduction in SES-CD, was assessed in 44 patients and achieved in 63.6% (28/44). This outcome was also significantly higher among AT-naïve patients (83.3% vs 50%; *P* = .049).

Multivariate analysis revealed higher baseline serum albumin was significantly associated with increased odds of remission (OR 1.73, 95% CI 1.15-3.46, *P* = .047), while higher haemoglobin (OR 1.13, 95% CI 1.02-1.32, *P* = .054), younger age at diagnosis (OR 1.11, 95% CI 1.01-1.29, *P* = .078), and shorter disease duration (OR 1.17, 95% CI 1.01-1.46, *P* = .090) demonstrated trends toward significance. Use of risankizumab as a second- or later-line therapy was independently associated with lower odds of achieving remission (OR 0.27, 95% CI 0.11-0.54, *P* = .001). In the AT-exposed subgroup, a multivariable analysis confirmed that prior ustekinumab exposure was significantly associated with lower odds of post induction clinical remission (OR 0.11, 95% CI 0.01-0.62, *P* = .021), while prior exposure to anti-TNF (*P* = .38), vedolizumab (*P* = .24), or upadacitinib (*P* > .9) was not significantly associated with this outcome.

Two patients with primary nonresponse after standard induction underwent extended induction with three additional intravenous 600 mg doses. One of these patients achieved delayed clinical response, while the other remained refractory. Additionally, eight patients underwent dose intensification to risankizumab every 4 weeks (Q4W) due to either suboptimal response or secondary loss of response during maintenance. Three of these eight patients received a 1200 mg IV rescue dose prior to dose intensification. Among these, five patients (62.5%) recaptured clinical response, of whom three entered clinical remission, during a minimum follow-up period of 6 months after dose intensification. To explore factors predicting the need for dose intensification, logistic regression analysis identified prior ustekinumab exposure as significantly associated with increased likelihood of escalation to Q4W dosing (OR 5.67, 95% CI 1.17-41.5, *P = *.045).

## Discussion

This prospective single-centre cohort study represents the first robust real-world evaluation to date of risankizumab in moderate-to-severe Crohn’s disease specifically from the Middle East, contributing crucial regional data to the growing global evidence on IL-23 inhibitors in IBD. The baseline phenotype reflects the aggressive disease increasingly seen in this region of the Middle East, with a high prevalence of stricturing, penetrating, and perianal complications—particularly among patients previously exposed to biologics or small molecules.^9^ Indeed, the rates of perianal disease (overall 32%, 43% in AT-exposed) and complex behaviours in our AT-exposed group underscore the challenging nature of CD in this cohort. These findings align with emerging regional data and highlight the need for early, effective intervention to alter disease progression in patients from this unique, under-explored demographic.

Despite the increased disease severity and complexity observed in our cohort compared to typical trial populations, the clinical outcomes broadly mirror the efficacy rates reported in pivotal trials. The ADVANCE/MOTIVATE induction trials and the FORTIFY maintenance trial established risankizumab’s efficacy, and our real-world remission and response rates, particularly among AT-naïve patients, were consistent with these findings within our Middle Eastern (predominantly Emirati) cohort.^4^^,^^5^ These results are encouraging and support the translation of clinical trial efficacy into real-world effectiveness, even in populations often underrepresented in global studies. Our findings also align with other real-world cohorts like the GETAID study, which showed similar induction effectiveness in refractory patients.^13^

Multivariable analysis identified several baseline predictors with potential clinical utility. Higher baseline serum albumin significantly predicted remission, possibly reflecting better inflammatory status or influencing pharmacokinetics, while trends for higher haemoglobin and shorter disease duration were also observed.^14^ The trend towards better outcomes with shorter disease duration echoes findings from PROFILE and risankizumab trial analyses, reinforcing the clinical implication that earlier use of effective therapies like risankizumab may improve long-term trajectories by intercepting disease progression before complications arise; a particularly relevant consideration in populations prone to aggressive CD phenotypes.^15^^,^^16^

A key finding with potential clinical relevance is observed association with prior therapy exposure. Our study confirms risankizumab’s effectiveness as a second-line agent after anti-TNF failure. However, prior ustekinumab exposure significantly reduced remission odds, similar to the GETAID cohort and other real world evidence studies, suggesting potential class-specific mechanistic implications or cross-resistance.^13^^,^^17^ This aligns with the SEQUENCE trial results favouring risankizumab over ustekinumab and some, though not all, real-world data.^18^ These findings underscore the importance of considering prior treatment history, particularly ustekinumab exposure, when positioning risankizumab in treatment algorithms, as it may influence expected outcomes, especially in complex patient populations from the Middle East.

Beyond initial efficacy, optimizing therapy for sustained response is critical. Our data support the clinical utility of dose optimization strategies. While standardized TDM isn’t established, higher drug levels correlate with better outcomes.^19^ We observed benefit from extended induction in one primary non-responder, aligning with clinical program data suggesting potential for delayed response with continued treatment.^20^ Furthermore, dose intensification to Q4W maintenance successfully recaptured response in 62.5% (5/8) of patients with suboptimal or loss of response, mirroring outcomes from other real-world cohorts.^21^ Our approach to dose optimization, particularly the use of a 1200 mg IV rescue dose prior to maintenance dose intensification, warrants further comment. While the IV rescue dose was aligned with the management strategy for inadequate responders in the FORTIFY maintenance trial, the subsequent dose intensification to Q4W was not part of the trial ­protocol.^22^^,^^23^ This strategy was instead an extrapolation from established real-world practice with ustekinumab, where IV re-induction followed by dose interval shortening is a common and effective method for recapturing clinical and endoscopic response in patients with secondary loss of response.^24^ Given the similar interleukin-targeting mechanisms, this approach was considered a rational clinical option at the time. Crucially, prior ustekinumab exposure was a factor significantly associated with needing dose escalation, further highlighting the practical implication that these patients may require closer monitoring and potentially earlier or more frequent dose adjustments to maintain response. In terms of safety, our data reaffirm risankizumab’s favourable profile. No serious infections, malignancies, or Crohn’s-related surgeries occurred during follow-up. The safety profile remained consistent even with dose intensification ­strategies, supporting their use when clinically indicated.

This study has several limitations. Although it represents the largest prospective real-world cohort of risankizumab-treated CD patients reported from the Middle East, the overall sample size remains modest, limiting subgroup analysis power. The observational design carries inherent risks of selection bias. Variability in the timing and availability of endoscopic/radiologic assessments is another limitation. Furthermore, the modest sample size constrains the statistical power of our multivariable regression models. The limited number of events relative to the covariates introduces a risk of overfitting, which is reflected in the wide confidence intervals and borderline statistical significance observed for several potential predictors. Therefore, these associations should be interpreted with caution and are best considered hypothesis-generating. Finally, longer-term follow-up beyond one year is needed to determine the durability of response and persistence. Nonetheless, this study provides valuable, contextually relevant evidence on risankizumab’s real-world performance in a unique, underrepresented Middle Eastern population, characterized by a high prevalence of aggressive CD. Our cohort, primarily Emirati nationals with complex CD, addresses a significant gap left by clinical trials. The findings robustly support risankizumab’s effectiveness and safety across different treatment lines and disease severities in this setting, informing its therapeutic positioning. Future research in the region should focus on longer-term outcomes, comparative effectiveness studies with other advanced therapies, and potentially exploring pharmacogenomic factors influencing response in MENA populations. These efforts are crucial for refining data-driven IBD treatment frameworks within the Middle East.

## Data Availability

Data supporting the findings of this study are available from the corresponding author upon reasonable request.
